# *iMAGING*: a novel automated system for malaria diagnosis by using artificial intelligence tools and a universal low-cost robotized microscope

**DOI:** 10.3389/fmicb.2023.1240936

**Published:** 2023-11-24

**Authors:** Carles Rubio Maturana, Allisson Dantas de Oliveira, Sergi Nadal, Francesc Zarzuela Serrat, Elena Sulleiro, Edurne Ruiz, Besim Bilalli, Anna Veiga, Mateu Espasa, Alberto Abelló, Tomàs Pumarola Suñé, Marta Segú, Daniel López-Codina, Elisa Sayrol Clols, Joan Joseph-Munné

**Affiliations:** ^1^Microbiology Department, Vall d’Hebron University Hospital, Vall d’Hebron Research Institute (VHIR), Barcelona, Spain; ^2^Department of Microbiology and Genetics, Universitat Autònoma de Barcelona (UAB), Barcelona, Spain; ^3^Computational Biology and Complex Systems Group, Physics Department, Universitat Politècnica de Catalunya (UPC), Castelldefels, Spain; ^4^Database Technologies and Information Group, Service and Information Systems Engineering Department, Universitat Politècnica de Catalunya (UPC), Barcelona, Spain; ^5^CIBERINFEC, ISCIII- CIBER de Enfermedades Infecciosas, Instituto de Salud Carlos III, Madrid, Spain; ^6^Probitas Foundation, Barcelona, Spain; ^7^Clinical Laboratories, Microbiology Department, Hospital Universitari Parc Taulí, Sabadell, Spain; ^8^Futbol Club Barcelona Foundation, Barcelona, Spain; ^9^Tecnocampus, Universitat Pompeu Fabra, Mataró, Spain

**Keywords:** malaria, malaria diagnosis, convolutional neural networks, artificial intelligence, robotized microscope, smartphone application, YOLOv5, thick blood smears

## Abstract

**Introduction:**

Malaria is one of the most prevalent infectious diseases in sub-Saharan Africa, with 247 million cases reported worldwide in 2021 according to the World Health Organization. Optical microscopy remains the gold standard technique for malaria diagnosis, however, it requires expertise, is time-consuming and difficult to reproduce. Therefore, new diagnostic techniques based on digital image analysis using artificial intelligence tools can improve diagnosis and help automate it.

**Methods:**

In this study, a dataset of 2571 labeled thick blood smear images were created. YOLOv5x, Faster R-CNN, SSD, and RetinaNet object detection neural networks were trained on the same dataset to evaluate their performance in *Plasmodium* parasite detection. Attention modules were applied and compared with YOLOv5x results. To automate the entire diagnostic process, a prototype of 3D-printed pieces was designed for the robotization of conventional optical microscopy, capable of auto-focusing the sample and tracking the entire slide.

**Results:**

Comparative analysis yielded a performance for YOLOv5x on a test set of 92.10% precision, 93.50% recall, 92.79% F-score, and 94.40% mAP0.5 for leukocyte, early and mature *Plasmodium* trophozoites overall detection. F-score values of each category were 99.0% for leukocytes, 88.6% for early trophozoites and 87.3% for mature trophozoites detection. Attention modules performance show non-significant statistical differences when compared to YOLOv5x original trained model. The predictive models were integrated into a smartphone-computer application for the purpose of image-based diagnostics in the laboratory. The system can perform a fully automated diagnosis by the auto-focus and X-Y movements of the robotized microscope, the CNN models trained for digital image analysis, and the smartphone device. The new prototype would determine whether a Giemsa-stained thick blood smear sample is positive/negative for *Plasmodium* infection and its parasite levels. The whole system was integrated into the *iMAGING* smartphone application.

**Conclusion:**

The coalescence of the fully-automated system via auto-focus and slide movements and the autonomous detection of *Plasmodium* parasites in digital images with a smartphone software and AI algorithms confers the prototype the optimal features to join the global effort against malaria, neglected tropical diseases and other infectious diseases.

## 1 Background

Malaria is a vector-borne disease caused by parasites of the genus *Plasmodium* (1). It is transmitted to humans by the bite of an infected female mosquito of the species *Anopheles* and has a high prevalence in tropical regions worldwide (Talapko et al., 2019). According to World Health Organization (WHO) estimates, 247 million cases of malaria were reported globally in 2021, increasing from 245 million in 2020 (World Health Organization, 2022). Malaria heavily affects the African Region with about 95% of all cases and 96% of all deaths (World Health Organization, 2022). There are five species of *Plasmodium* parasites that can infect humans: *P. falciparum*, *P. vivax*, *P. ovale*, *P. malariae*, and *P. knowlesi* (Talapko et al., 2019).

Microscopic visualization of blood smears is still the gold-standard method for malaria diagnosis (Calderaro et al., 2021). The examination of thick and thin blood smear samples by traditional optical microscopy allows the visualization of active parasitic forms in peripheral blood (WHO, 2022). Differentiation of parasite species is performed by the visualization of thin blood smears with Giemsa staining (WHO, 2016). Thick blood smear is a 20-40-fold more sensitive technique compared with thin smears and, therefore, can visualize lower parasite levels (Wangai et al., 2011). Microscopy is an inexpensive and efficient technique that allows the identification of *Plasmodium* parasites at the species level, the determination of different developmental stages of the parasites, and the quantification of parasite density. It is widely used in endemic and low-income areas from Sub-Saharan Africa and reference laboratories worldwide. However, it is an expert-dependent technique, can generate diagnostic mistakes due to the consecutive visualization of a high number of samples, and can lead to diagnostic inaccuracies due to fatigue and workload.

Rapid Diagnostic Tests (RDTs) are blood antigen detecting tests with an immunochromatographic lateral flow device that allows the diagnosis of malaria parasites (Cunningham et al., 2019). The limit of detection of malaria RDTs is about 100–200 parasites/μL of blood (Berzosa et al., 2018; Acquah et al., 2021). However, the emergence of gene deletions coding for the HRP2/3 proteins is causing an increase in false-negative results due to the lack of the detection antigen with consequences for the final diagnosis (Jejaw Zeleke et al., 2022). Polymerase Chain Reaction (PCR) for malaria diagnosis (i) is a highly sensitive technique (Leski et al., 2020); (ii) allows to distinguish between *Plasmodium* species (van Bergen et al., 2021); (iii) requires specific material, and (iv) is costly and relatively complex (Fitri et al., 2022). Quantitative Buffy Coat (QBC), Flow cytometry, or biomarker identification are used to complement traditional methods (Calderaro et al., 2021). Nevertheless, malaria diagnosis is still an issue in some regions, which could lead to misdiagnosis and generate several complications due to the difficulty of implementing these techniques in resource-poor settings (Boyce and O’Meara, 2017). The lack of resources and health care personnel in malaria-endemic areas are a limitation for accurate diagnosis (Wambani and Okoth, 2022). Moreover, traditional diagnostic techniques are time-consuming and require high-level trained professionals. Thus, the development of accessible, low-cost, automated diagnostic techniques is a major challenge for malaria parasite detection and would be a supportive complement to traditional techniques.

Nowadays, Artificial Intelligence (AI) is a disruptive technology with a high impact on health-related goals. Convolutional Neural Networks (CNNs) are artificial neural network models commonly used to analyze and classify images with deep learning tools; and they have improved traditional image-processing techniques through their faster and highly automated procedure. Novel diagnostic techniques based on AI are being developed and optimized for the detection of *Plasmodium* parasites in thick and thin blood smear digital images (Sankaran et al., 2017; Fatima and Farid, 2020; Yang et al., 2020; Abubakar et al., 2021; Islam et al., 2022). CNN-based malaria detection algorithms able to detect *P. falciparum* parasites in Giemsa stained thick blood smear slides were developed and demonstrate robustness with a wide variety of field-prepared samples (Mehanian et al., 2017a,b). As another example, DeepMCNN system was able to calculate parasitaemia estimations by counting parasites and leukocytes as recommended by the WHO (Manescu et al., 2020). Algorithm analysis is a crucial step to correctly evaluate and implement machine learning solutions for clinical usage with effective metrics (Delahunt et al., 2022).

Mobile software applications are used to integrate the technology and provide a fast and efficient diagnosis for *Plasmodium* detection (Rosado et al., 2016; Vasiman et al., 2019). However, smartphones have several limitations that should be addressed such as: image resolution required for malaria diagnosis, optical attachment and adaptation to the microscope, high number of fields-of-view (FoVs) for diagnosis, and the need for focused images and Z-stacks (Mehanian et al., 2017a,b; Delahunt et al., 2022).

Moreover, automation of the entire process, including autofocusing and slide tracking movements, would be of significant help to obtain an optimal diagnostic tool. Robotized microscopes that move the slide and capture images are usually expensive and not designed for implementation in malaria-endemic countries. Observational studies to evaluate the performance of automated detection systems for malaria diagnosis were performed in in-field settings with promising results, such as EasyScan Go and Autoscope (Torres et al., 2018; Horning et al., 2021; Das et al., 2022). This type of device could be a useful diagnostic tool, not only for malaria diagnosis, but also for other infectious or Neglected Tropical Diseases (NTDs), such as schistosomiasis, trypanosomiasis, and filariasis. However, specific peculiarities in terms of optical train, number of FoVs for diagnosis, parasite sizes and sample preparation should be considered.

In this study, we trained multiple computational state-of-the-art deep learning models for malaria parasite detection in thick blood smear digital images. A malaria-labeled image database was created and employed to train the CNN models. We compared the performance of different neural networks with the same dataset, and applied attention modules. In addition, a low-cost robotized microscope was designed to automate the image auto-focus and slide movements. Arduino controllers, 3D-printed pieces, and servo motors were used to create a single prototype for the universal automation of optical microscopes. The system does not need internet connection and was power supplied by portable solar batteries. Finally, the diagnosis technology has been integrated into a smartphone application called *iMAGING*, which controls the microscope’s slide movements and detects malaria parasites in digital images via trained CNNs on a computer. According to our knowledge, it is the first fully automated low-cost system for malaria diagnosis with artificial intelligence tools, and specially designed for low-income regions and malaria endemic regions. We consider that the development of this novel digital image diagnosis technology would contribute to erradicate malaria and other neglected tropical diseases, and would join the global effort to fight against infectious diseases of poverty.

## 2 Materials and methods

### 2.1 Convolutional neural network algorithms

**Figure 1:**
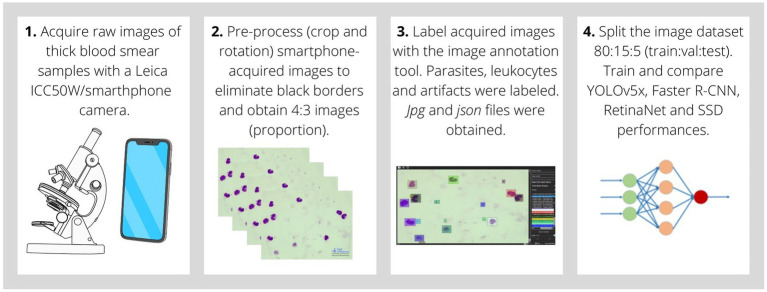
Sequential procedure of CNN algorithm generation for malaria parasite detection in thick blood smear digital images. **1.** Images were acquired through the microscope lens with an ICC50W integrated digital camera or a smartphone camera. **2.** Smartphone-acquired images were pre-processed to eliminate black borders from the surroundings of the original raw image by image cropping and rotation. New images have a 4:3 proportion, emulating integrated camera-acquired images. **3.** Image labeling by image annotation software. Parasites, leukocytes, and artifacts were labeled for further CNN training. JPG (image) and JSON (labels linked to image) files were generated. **4.** Split image dataset into 80% training, 15% validation, and 5% testing. Train and compare YOLOv5x, Faster R-CNN, RetinaNet, and SSD performances.

To generate the malaria digital image database and train CNN algorithms, the following methodologies, summarized in Figure 1, were employed. The same dataset was used to train the different CNN models. This methodology follows quality standards and was the same for all experiments. This type of work allows us to standardize the methods thus obtaining reliable and comparable results between the neural network models employed.

#### 2.1.1 Image acquisition

Giemsa-stained thick blood smear samples were used to capture microscopic digital images for further image labeling and identification. Thick blood smear biological samples were provided by the (i) Vall d’Hebron University Hospital (Barcelona, Spain), (ii) Drassanes-Vall d’Hebron International Health and Infectious Diseases Centre (Barcelona, Spain), (iii) Malaria collection samples of Drassanes-Vall d’Hebron, and (iv) Saint John of God Hospital (Lunsar, Sierra Leona). Parasites of *P. falciparum*, *P. vivax*, *P. ovale*, and *P. malariae* species were visualized in the samples used to correctly detect *Plasmodium* infection. Samples were validated by three expert clinical parasitology microscopists from Drassanes-Vall d’Hebron International Health and Infectious Diseases Centre Laboratory. All thick blood smear samples were positive for *Plasmodium* infection with parasite levels ranging from 80 to +10,000 parasites/μL. A Leica ICC50W integrated digital microscopy camera (5.0 MP) and the digital camera of a Samsung Galaxy S20 (64 MP, 0.8 μm, f/2.0, OIS) smartphone device were employed for image acquisition. Image pixel size (resolution) was 3,024 × 4,032 pixels for smartphone-captured images, and 2,992 × 1944 pixels for the Leica ICC50W digital camera. An adapter 3D bracket attached to the ocular lens of the microscope was used to standardize the image-capturing procedure with the smartphone device. Both, integrated camera and smartphone images were captured by the visualization of blood smear samples through a Leica DM750 microscope lens with 1,000x total magnification (10x ocular; 100x immersion oil objective).

#### 2.1.2 Image pre-processing

The images were cropped to highlight the area of interest and eliminate the black borders typical of acquisition with a smartphone device. Cropping was only performed in smartphone-acquired images to remove the outer edges without losing information. Original smartphone images were cropped automatically (Python script) to obtain a 4:3 image in the center, and subsequently rotated 90° for image reorientation. With this procedure, it is possible to crop an image regardless of its dimensions and number of pixels, as proportions were used to perform the cropping. The cropped images have the same 4:3 image proportion as those acquired with the microscope-integrated camera. Cropping confers a recomposition of the image that may positively affect the final results, providing a clearer image and removing elements irrelevant to the prediction and identification functions of the neural networks (Cheng et al., 2022). Pre-processed images were used for image annotation and CNN training.

#### 2.1.3 Image annotation

Both, integrated digital camera and smartphone camera acquired images were labeled by experts of the Drassanes-Vall d’Hebron International Health and Infectious Diseases Centre. Parasite forms, leukocytes, and artifacts were labeled in malaria thick blood smear digital images. A personalized annotation software was developed with the Python programming language for digital image labeling (Python Software Foundation, 2019). For image annotation, the area of interest was selected by creating a bounding box with the object inside. Labels were considered single-object detections on digital database images, therefore an image contains multiple annotations. Parasite forms (malaria blood stage cycle) as early trophozoites and mature trophozoites were labeled. Leukocytes were annotated in digital malaria images for further parasite density calculations. Artifacts and confusing forms due to illumination issues, sample preparation, Giemsa staining reagents, or image capturing were also labeled. The annotation procedure is represented in Figure 1. Once labeling is finished, the Annotation App software creates a *json* file with annotations linked to the original image file, in which the coordinates of the labeled objects are specified.

#### 2.1.4 Convolutional neural networks training

A comprehensive comparative study to evaluate the performance of several state-of-the-art object-detection CNN models was designed. The comparison of the convolutional neural networks has been designed based on previous similar studies (Dong et al., 2017; Pattanaik et al., 2019). Pre-trained YOLOv5 (Redmon et al., 2016), Faster R-CNN (Ren et al., 2017), SSD (Liu et al., 2016), and RetinaNet (Pardede et al., 2020) models with the COCO dataset (Dataset, n.d.) were fine-tuned with our malaria thick blood smear positive *Plasmodium* infection samples dataset. CNNs were trained for multi-class classification with three categories: early trophozoites, mature trophozoites, and leukocytes. Their performances were evaluated by precision, recall, *F*-score, and mAP0.5 descriptive values with validation and test sets. The malaria dataset was split into the same proportions for each CNN model: 80% images for training, 15% for validation and 5% for testing. Images/samples for each subset were the same to standardize and compare performances under equal training conditions, preserving patient-level structure.

#### 2.1.5 Attention modules

Preliminary tests to check attention modules perfomance in our malaria dataset with the same proportion split (80% train / 15% validation / 5% test) were carried out. In particular, we have trained the YOLOv5x with the Squeeze and Excitation (SE) attention module, as well as the Convolutional Block Attention Module (CBAM) as stated in Hu et al. (2018) and Woo et al. (2018) respectively. Their performances were evaluated by precision, recall, *F*-score, and mAP0.5 descriptive values with test set and compared with YOLOv5x results as demonstrated in other studies (Gong et al., 2022).

#### 2.1.6 Statistical analysis

Statistical analyses to determine significant differences between validation and test subset performance for each CNN model was performed. Metric means were calculated individually for each CNN model and subset (validation/test) by t-test analysis (*p* < 0.05). To evaluate significant statistical differences between CNNs models and attention modules a paired *t*-test analyses (*p* < 0.05, *t*-value > 2/−2), mean (M) and standard deviation (SD) were employed. Statistical analyses were performed with IBM SPSS Statistics environment.

### 2.2 Microscope automation and smartphone application

#### 2.2.1 Design of a 3D-printed prototype for microscopy automation

An Ender-3 Creality 3D printer was used to build Polylactic Acid (PLA) and Polyethylene terephthalate glycol (PETG) pieces for microscope focus automation. The entire prototype was designed with Tinkercad Open Source specialized software and Ultimaker Cura software (Ultimaker Cura: software de impresión 3D potente y fácil de usar | Ultimaker, n.d.). Autofocusing and two-dimensional *X*-*Y* track slide movements were performed by low-cost servo and step-by-step motors. Power requirements are: servo motor 9G / 5 V and 500 mA each; stepper motor Rohs 28BYJ-48 / 5 V and 240 mA; and Arduino MKR Wifi 1,010 / 5 V and 700 Ma. The whole system requirements are 5 V and 2A.

#### 2.2.2 Auto-focus algorithm

In our mechanism we have employed the variance of Laplacian as a reference method for image auto-focusing. This method allows the calculation of a value for each image, which indicates the level of focusing of the acquired picture. Therefore, the analysis of variance of Laplacian values would determine which is the best focused image for each Field of View (FoV) (Salido et al., 2020). The variance calculation is performed in each FoV determined by *X*-*Y* movements of the robotized microscope. In a single FoV the smartphone camera observes different focused images by the continuous movement of the step-by-step motor on the fine adjustment wheel. The smartphone device by Bluetooth (*BLE*) connection with the controllers guides the step-by-step motor to move the wheel in both directions of rotation (30 position units of movement in each direction) in order to focus the biological sample. During the auto-focusing process a Laplacian variance value is computed to each of the images. The system visualizes the centroid of the original image by creating a new cropped image for Laplacian analysis. This procedure allows the observation of only the center of the image, without the black borders produced by the ocular lens attachment. Once the image scanning in the two directions of rotation has been completed, the mechanism is able to return to the position of highest focus by analyzing the variance values. An image was captured in the focused position by the smartphone camera for further image analysis.

#### 2.2.3 Integration of trained CNN models into computer software

A Lenovo ThinkPad intel Core i5 computer was employed to run CNN algorithms. A smartphone app was developed with the official open-access integrated development environment, Android Studio (Android Developers, 2021). The *iMAGING* smartphone app confers the possibility of integrating CNN and automated microscope technology into a single software. To increase computational power and speed up neural network detection, images captured from the smartphone device are sent via a *BLE* connection to the computer for further analysis by CNN models. This type of transmission does not depend on an internet connection.

## 3 Results

**Figure 2:**
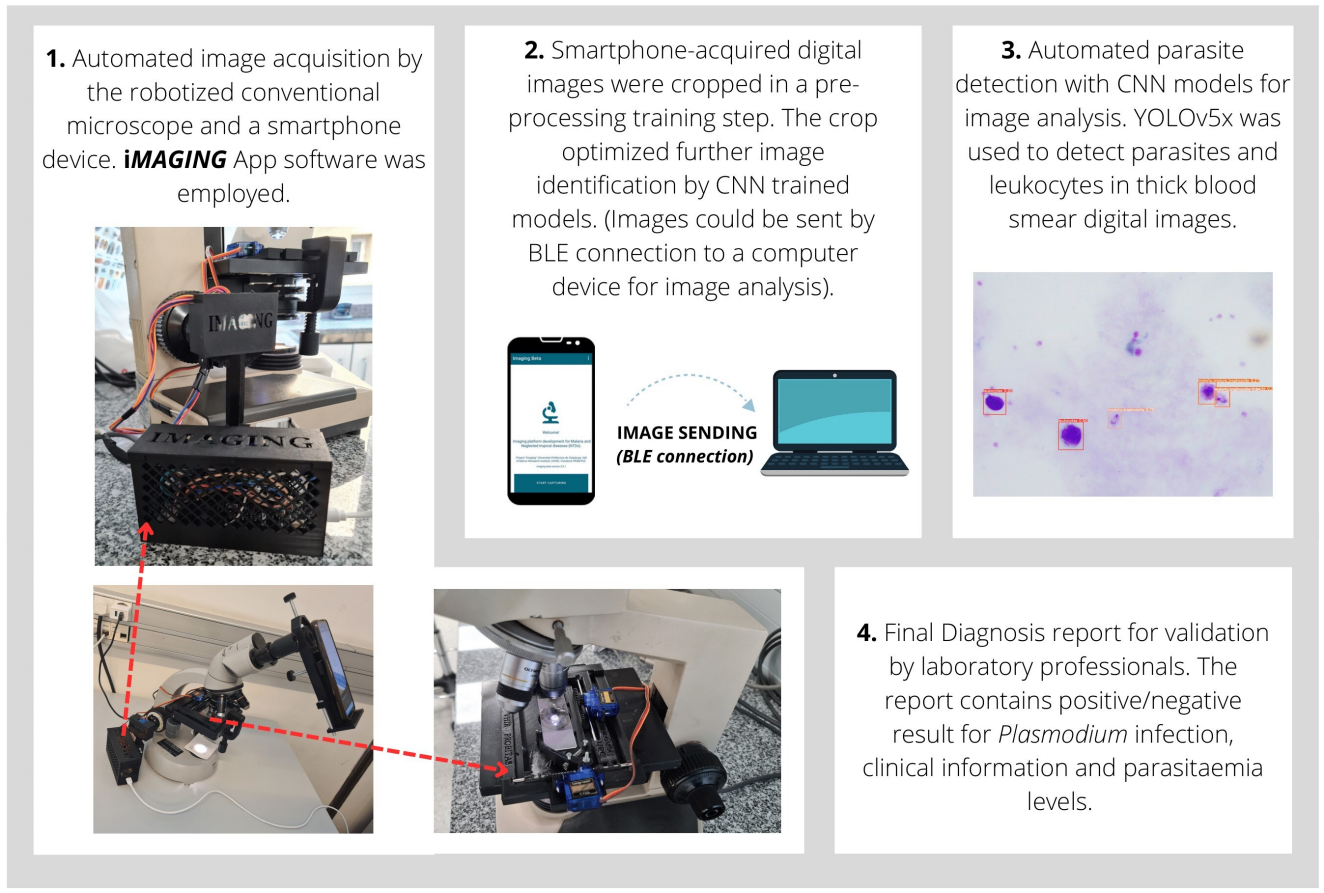
Flowchart of the malaria diagnostics procedure by *iMAGING* technology. The robotized microscope and smartphone were used as an emulation of traditional microscopic examination. **1.** The smartphone captures images via its camera using the *iMAGING* app. Images of the system prototype were represented. **2.** Images are cropped to eliminate areas of no interest for imaging diagnosis. Images are sent by a Bluetooth (*BLE*) connection to a computer for further analysis. **3.** Images are analyzed by computational technology and Convolutional Neural Network models for parasite detection. **4.** Finally a malaria diagnosis report containing clinical information is generated.

A novel automated diagnostic method for malaria parasite detection in thick blood smear digital images was developed and integrated into a smartphone app. The flowchart of the operational procedure is represented in Figure 2.

### 3.1 Convolutional neural network

#### 3.1.1 Malaria thick blood smear image database analysis

Once all images were captured and labeled, a digital image database of malaria thick blood smears was generated; a total of 148 thick blood smear samples were used for image database generation. Between 10 and 20 images of different microscopy fields were captured for each sample employed. A total of 2,571 labeled images were imported into the database for further CNN training and diagnosis algorithm generation. Annotation numbers for each label class were: 24437 leukocytes, 37,820 early trophozoites and 1,641 mature trophozoites were labeled taking into account all database images. A total of 2,238 images were captured with the integrated microscope camera LEICA ICC50W and 333 images were acquired with the Samsung Galaxy S20 smartphone camera. Malaria thick blood smear image database summary information is represented in Table 1. The total number of images and labels employed for CNN training was decided considering other similar studies (Mehanian et al., 2017a,b; Torres et al., 2018; Yang et al., 2020), and were conditioned by the number of *Plasmodium* infection samples available in the laboratory and CNN algorithms proven performance results.

**Table 1:** Summary of Malaria thick blood smear sample/image database.

Category		Sub-Total	Total
Sample source	Drassanes-Vall d’Hebron International Health and Infectious Diseases Centre (Barcelona, Spain)	55 (samples/patients)	148 (samples/patients)
	Malaria collection samples of Drassanes-Vall d’Hebron (Barcelona, Spain)	82 (samples/patients)	
	Saint John of God Hospital (Lunsar, Sierra Leona)	13 (samples/patients)	
*Plasmodium* species	*P. falciparum*	47 (samples)	80 (samples)
	*P. vivax/P. ovale*	24 (samples)	
	*P. malariae*	7 (samples)	
	Mixed infection	2 (samples)	
Parasite density	Low (<1,000 p/μL)	57 (samples)	148 (samples)
	Medium (10,000–1,000 p/μL)	57 (samples)	
	High (+10,000 p/μL)	34 (samples)	
Image acquisition type	Integrated camera (ICC50W Leica)	2,238 (annotated images)	2,571 (annotated images)
	Smartphone (Samsung Galaxy s20)	333 (annotated images)	
Annotations category	Early trophozoites	37,820 (labels)	63,898 (labels)
	Mature trophozoites	1641 (labels)	
	Leukocytes	24,437 (labels)	

p, parasites; μL, microliter. Organizational database scale: Patient > Sample > Annotated Image > Annotation.

#### 3.1.2 Convolutional neural network comparison

Object-detection state-of-the-art CNNs were trained and compared to evaluate their performance. Table 2 shows the most relevant metrics to evaluate the performance of YOLOv5x, Faster R-CNN, SDD, and RetinaNet with validation and test data image subsets. Results of the t-test analysis indicated that there was a non-significant statistical difference between neural network performance with validation and test data subsets, as expected (Table 2).

**Table 2:** Comparative table of object-detection CNN models’ performance.

CNN model	Validation dataset				Test dataset				
	Precision	Recall	*F*-score	mAP 0.5	Precision	Recall	*F*-score	mAP0.5	*p*-value
YOLOv5x	0.8975	0.9197	**0.9085**	**0.9490**	0.9210	0.9350	**0.9279**	0.9440	0.157
Faster R-CNN	0.8753	**0.9331**	0.9033	0.9194	0.8913	**0.9638**	0.9261	0.9412	0.144
SSD	**0.9501**	0.4789	0.6368	0.8491	**0.9562**	0.5599	0.7063	0.9133	0.354
RetinaNet	0.9369	0.8155	0.8720	0.9180	0.9407	0.8719	0.9050	**0.9489**	0.187

Descriptive parameter values of Precision, Recall, *F*-score, and Mean Average Precision (mAP0.5) are represented for Validation and Test datasets. YOLOv5x: You Only Look Once version 5 model x, Faster R-CNN: Faster R-Convolutional Neural Network, SSD: Single Shot Detector. Statistical analysis (*t*-test) to compare the performance of CNN models with validation and test data subsets (*p* < 0.05). Bold values represent the higher values of each parameter, in validation and test datasets.

Precision test values ranged between 0.8913–0.9562, setting considerably optimized results with all trained CNN models. Precision parameter analysis indicates that all CNNs have an optimized identification algorithm, with low rates of failures when parasite and leukocyte detections were performed. Analysis of recall (sensitivity) test values generated a wide range of values: 0.4789–0.9331. These remarkable differences between CNNs indicate that YOLOv5x and Faster R-CNN, with recall values of 0.9350 and 0.9638, respectively, were the optimal CNNs for these type of detections. Low recall values, as for SSD, indicate that the algorithm could not detect all objects of interest and, therefore, high levels of false-negative results were obtained.

The *F*-score is the harmonic mean of precision and recall (Equation 1); therefore, *F*-score analysis of the different trained CNNs was relevant to determine the model with the best performance. The highest *F*-score value was 0.9279, corresponding to the YOLOv5x neural network. The Faster R-CNN *F*-score value was just lower than that of YOLOv5x, with a final result of 0.9261. Both neural networks were optimal in terms of accuracy and, therefore, with high precision and recall values. In Supplementary Figure 1, the precision-recall graph of the YOLOv5x algorithm demonstrates the aforementioned results.


(1) $$F\;score=2\;\cdot \;\frac{precision\;\cdot \;recall}{precision+recall}$$

Equation (1): *F*-score value calculation.

Finally, mAP values were between 0.9133–0.9489, indicating a high accuracy value for the trained object-detection models.

**Figure 3:**
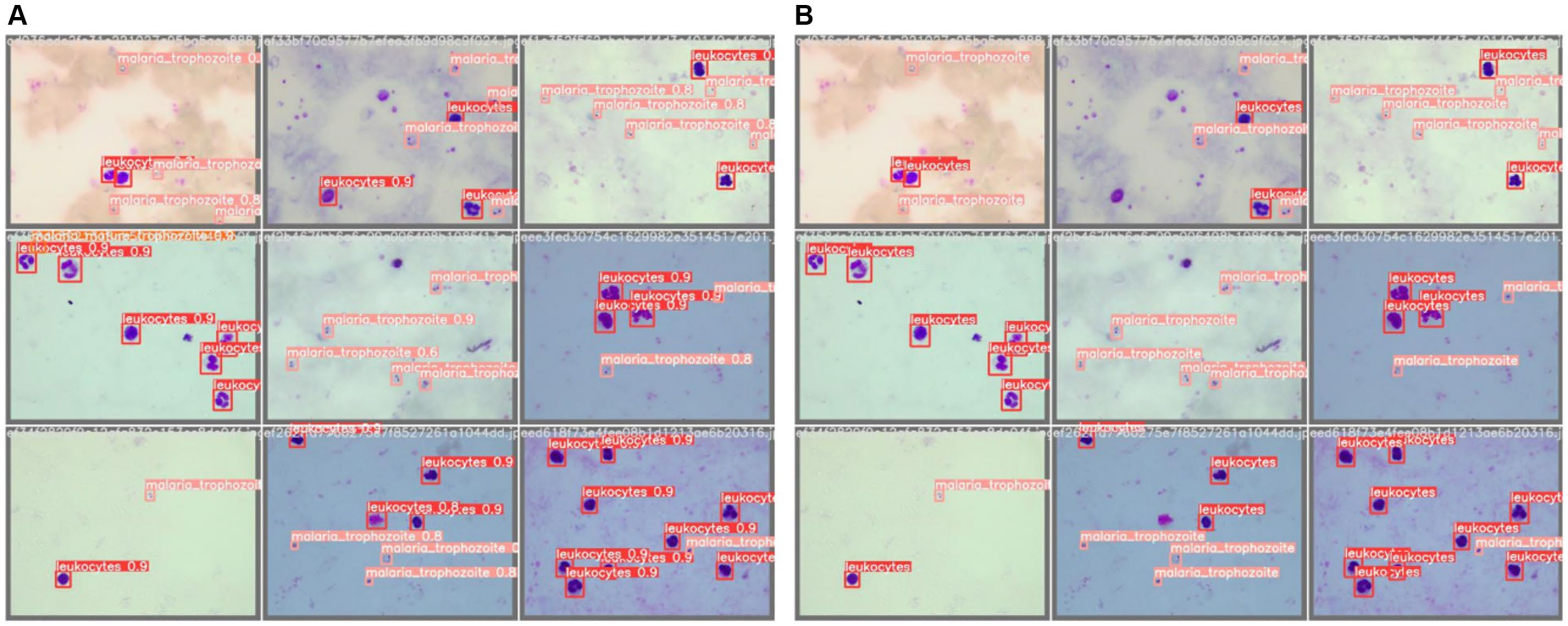
**(A)** Labeled test images by Vall d’Hebron-Drassanes professionals. **(B)** YOLOv5x trained model predictions of a test image subset with confidence values.

Once all descriptive parameters were analyzed, we concluded that YOLOv5x and Faster R-CNN were the best CNNs based on object detection for our image database. Results of the paired t-test indicated that there was a non-significant difference between YOLOv5x (*M* = 0.9; *SD* = 0.02) and Faster R-CNN (*M* = 0.9; *SD* = 0.03), *t*(7) = 0.8, *p* = 0.429. The low recall values for the SSD model indicate a non-reliable algorithm for parasite detection. The RetinaNet model had acceptable results, although not as positive as YOLOv5x and Faster R-CNN. As a result, we present several digital labeled images of a test data subset in Figure 3, and predictions performed by the YOLOv5x trained model.

Finally, in order to evaluate the multi-class model for each category, results of YOLOv5x were shown in Table 3. Descriptive parameters of each class are important to evaluate the model for each category. Leukocytes have higher predictive values than early and mature trophozoites for all descriptive metrics.

**Table 3:** YOLOv5x model parameters for each label classification category.

Category	Precision	Recall	*F*-score	mAP0.5
Leukocytes	0.989	0.993	0.990	0.994
Early *Plasmodium* trophozoites	0.908	0.866	0.886	0.938
Mature *Plasmodium* trophozoites	0.838	0.912	0.873	0.905

#### 3.1.3 Attention modules comparison

Attention modules results of SE and CBAM training with YOLOv5x algorithm are shown in Supplementary Table 1. Precision values of CBAM (0.9350) are slightly better when compared with original YOLOv5x (0.9210) and SE (0.9040). In the particular case of recall values, SE showed the best performance (0.9380) just higher than that of YOLOv5x (0.9350). Therefore, *F*-score and mAP0.5 values of attention modules were similar to the original YOLOv5x trained model. Results of the paired t-test analysis indicated that there is a non-significant difference between YOLOv5x (*M* = 0.9, *SD* = 0.01) and CBAM (*M* = 0.9, *SD* = 0.01), *t*(3) = 0.3, *p* = 0.779; and that there is a non-significant difference between YOLO (*M* = 0.9, *SD* = 0.01) and SE (*M* = 0.9, *SD* = 0.02), *t*(3) = 1.1, *p* = 0.348. These results lead us to conclude that the application of attention modules in our database could help to obtain comparable results, although they did not improve on the performance of the original YOLOv5x CNN.

#### 3.1.4 YOLOv5x negative sample validation

Analysis and validation tests were performed to evaluate the reliability of YOLOv5x model with confirmed negative samples. Thick blood smear samples from asymptomatic individuals (healthy control) from malaria endemic areas were (n = 5) provided by the Drassanes-Vall d’Hebron International Health and Infectious Diseases Centre with a negative RT-PCR result for *Plasmodium* infection (RealStar^®^ Malaria Screen & Type PCR kit 1.0, Altona, 22,767 Hamburg, Germany). A total of 166 images were acquired from thick blood smear samples and analyzed by the trained YOLOv5x model (confidence threshold 0.5). The employed images were not used for CNN algorithms training. A minimum number of 200 leukocytes were detected in each sample to emulate microscopy examination. After analysis, 161/166 digital images (96.98%) were negative for *Plasmodium* infection, and five false-positive results were reported (five artifacts detected as early trophozoites). A total of 1,008 leukocytes were detected in all digital images. Validation tests were crucial to evaluate the reliability of the system and illustrate the importance of clinical validation by professionals to report a final diagnosis. Inspection by laboratory professionals is desirable to conclude that the sample was negative and discard false-positive results.

#### 3.1.5 YOLOv5x Low-parasite density validation

Analysis and validation tests were performed to evaluate the reliability of YOLOv5x model with confirmed low parasite density *Plasmodium* infected samples. Thick blood smear samples (*n* = 5) provided by the Drassanes-Vall d’Hebron International Health and Infectious Diseases Centre with a positive microscopy examination result (<800 parasites/μL) for *Plasmodium* infection were analyzed (confidence threshold 0.5). A total number of 50 *Plasmodium* positive images (10 images/sample) were acquired. Low-parasite images with a single *Plasmodium* parasite were analyzed to detect false-negative results. 47/50 images (94%) were correctly analyzed, with a total of three false-negative results (undetected mature trophozoites in a single sample). Final validation by laboratory professionals is desirable to conclude that the sample was positive and discard false-negative results.

#### 3.1.6 Automated parasite density calculations

The parasite level calculation procedure was based on the Centers for Disease Control and Prevention (CDC) recommendations for parasite density calculation in thick blood smear samples (CDC – DPDx – Diagnostic Procedures – Blood Specimens, n.d.). The system can acquire images and detect the number of parasites and leukocytes in each microscopic field. Once the system has quantified 200 leukocytes, a parasite density result is obtained. The number of *Plasmodium* parasites and leukocytes was simultaneously counted to obtain a quantitative result. The CDC estimates that 1 μL of blood contains 8,000 leukocytes (CDC – DPDx – Diagnostic Procedures – Blood Specimens, n.d.), therefore, an automatic calculation of parasite number per microliter could be performed. In cases where less than 200 leukocytes were counted, an approximate value would be obtained with the same calculation. Equation (2) represents the algorithm for automatic parasite density calculation by image analysis.


(2) $$\mathrm{Parasite}\;\mathrm{density}=\frac{\left(NDP:\mathrm{number}\;\mathrm{of}\;\mathrm{detected}\;\mathrm{parasites}\right)*\left(8,000\;\mathrm{leukocytes}\right)}{\left(NDL:\mathrm{number}\;\mathrm{of}\;\mathrm{detected}\;\mathrm{leukocytes}\geq 200\right)}=\mathrm{parasites}/\mathrm{\mu L}$$

Equation 2: Parasite density calculation in thick blood smear samples by image analysis. The number of *Plasmodium* parasites and leukocytes detected in several digital images would determine the number of parasites per microliter of blood. A total number of 8,000 leukocytes were assumed in 1 μL of peripheral blood.

### 3.2 Microscopy automation and smartphone application

#### 3.2.1 Universal microscopy automation prototype

A low-cost prototype was designed to automatically capture and detect malaria parasites in thick blood smear images on a conventional optical microscope. The main aim of microscope automation was to move the *X*-*Y* axis of the microscope stage and the auto-focus of the sample.

For the *X*-*Y* axis, an adapter was constructed to fit onto the microscope stage and hold the sample easily. Two servo-motors, one for each axis, capable of horizontal and vertical movements by means of toothed rotors, were attached to the same part. These pieces allow the movements across the sample, thus emulating the tracking of conventional microscopy. The *X*-*Y* control of the slide movement allows microscopic images to be captured for malaria diagnosis without overlapping each other. Each digital image corresponds to a microscopic field. The servo motors are controlled by Arduino controllers and connected via Bluetooth (version 5.0) to the smartphone device.

A step-by-step motor with an adapter arm was used for sample auto-focus. This was also monitored by Arduino controllers and connected via *BLE* to the smartphone device. For sample autofocusing the Variance of Laplacian has been employed (Salido et al., 2020). Additionally, an external cage was designed to store Arduino controllers, cables, and electronic parts. All 3D parts were designed as generic adapters by using measurements from various conventional optical microscopes commonly employed in laboratories (see Figure 2).

The motors used were finally controlled by a smartphone device, which captures microscopic images of different microscopy fields to detect biological forms in digital images. Image-capturing optimization was crucial for correct image acquisition. A smartphone adapter was designed to correctly attach the mobile phone lens to the ocular lens.

The whole 3D prototype was able to move the sample on the microscope stage, auto-focus the preparation, and capture digital images by means of a smartphone device. This mechanism allows automation of malaria diagnosis by the observation of thick blood smears and detection of parasite forms, thus emulating conventional microscopy. The smartphone device, step-by-step and servo motors, and controllers are powered by portable solar batteries.

#### 3.2.2 Autofocus evaluation

An auto-focus experiment was performed to calculate the time of focus and prediction time of YOLOv5x CNN. A total number of 50 FoV of thick blood smear samples were auto-focused and analyzed by *iMAGING* prototype system. Results show an average time of focus of 8,144 ± 56 ms/FoV; and an average analysis time by YOLOv5x CNN and a Lenovo ThinkPad intel Core i5 of 2,126 ± 179 ms/image. Analyzed images show 6.2 ± 2.3 leukocytes/image and 7.1 ± 2.7 trophozoites/image (ring stange and mature), with a total of 13.3 ± 3.9 objects/image.

#### 3.2.3 Imaging app software for malaria diagnosis

The imaging app software for smartphone devices was developed using the Android Studio development environment (Android Developers, 2021). The application allows the integration of all the technology developed for autonomous diagnosis. The smartphone, via the application, could control and guide the movements of the microscope slide via a *BLE v5.0* connection and capture digital images in each microscopic field. Therefore, the mobile device acts as the key element of the process and is responsible for capturing the images that will subsequently be analyzed by the trained CNN models on the computer. Mobile phone devices are relatively low-cost and available worldwide; for this reason, they are ideal for imaging diagnostics and might be a suitable option for implementation in areas with resource-poor settings.

## 4 Discussion

CNNs have been employed for the detection of *Plasmodium* parasites in thick and thin blood smear microscopic images. However, whole procedure automation, huge labeled image datasets, system implementation in clinical laboratories and effectiveness are some of the major issues of these technologies.

To address some of the main limitations, we have developed a fully automated diagnostic system for the detection of *Plasmodium* trophozoites and leukocytes in thick blood smear digital images by using AI tools and a low-cost robotized microscope. Training results showed optimal performance for early trophozoite, mature trophozoite, and leukocyte detection in a test dataset with the YOLOv5x and Faster R-CNN algorithms. Moreover, the adaptor 3D pieces confer the microscope the possibility to auto-focus the sample, scan the entire slide, and capture digital images with a smartphone camera for further image analysis and diagnosis. However, the system still has some limitations: (i) the need of trained personnel to prepare the Giemsa stain; (ii) it is able to detect *Plasmodium* spp. infection for malaria diagnosis, although it is not able to differentiate between *Plasmodium* species; and (iii) the difficulties that might appear in the field for its routinely implementation.

In our study, thick blood smears were used as reference samples for *Plasmodium* parasite detection. Thick blood smears should be the first step in microscopic visualization for malaria parasite detection, resulting in a positive/negative result for the sample analyzed (Maturana et al., 2022). Its observation is crucial to perform a malaria diagnosis and is widely employed in resource-poor settings due to its accessibility, conferring a valuable feature to the system. The detection of *Plasmodium* parasites and leukocytes by CNN algorithms provides a fast and efficient diagnosis. Moreover, species identification should be complemented with thin blood smear visualization for a complete diagnosis. Several studies demonstrate the possibility of detecting malaria parasites in thick (Kaewkamnerd et al., 2012; Rosado et al., 2016; Yang et al., 2020) and thin blood smear samples (Mushabe et al., 2013; Fatima and Farid, 2020), although few distinguish between species. The major limitation for *Plasmodium* species differentiation is the morphological similarities between them such as *P. ovale* and *P. vivax* (CDC, 2013), and the large image database required for the proper development of algorithms capable of differentiating between species (Krishnadas et al., 2022). Differentiation between *P. falciparum* and *P. vivax* could be the first step toward the generation of new models capable of detecting malaria parasites at the species level (Penas et al., 2017).

Moreover, the creation of a large database of thick blood smear labeled images (a total number of 63,898 labels) by professionals from an international health reference center confers an additional value to the diagnosis system. The employment of clinical biological samples is of vital importance for the acquisition and labeling of digital images as they more accurately emulate the practice of a microbiological diagnostic laboratory. In this study, there was an imbalance in label proportions between parasite stage forms due to biological reasons and the type of samples used. When thick blood smears were observed for *Plasmodium* parasite detection, the most common forms were early trophozoites or immature trophozoites (Phillips et al., 2017). In addition, most samples employed for database generation contain *P. falciparum* parasites, in which the majority of parasitic forms in peripheral blood are early trophozoites. Therefore, YOLOv5x, Faster R-CNN, SDD, and RetinaNet CNN models were trained with leukocyte, early trophozoite, and mature trophozoite image data labels. Generated models can detect the most common parasitic forms in thick blood smear samples and count leukocyte numbers to calculate parasite levels. Thus, trained CNNs could determine whether a sample is positive or negative for *Plasmodium* infection. *F*-score values are an optimal descriptive metric to evaluate or determine the best CNN model in cases of unbalanced data (Lopez-Nava et al., 2020). In cases of low-parasite levels a laboratory expert would be required to determine if the sample is positive/negative for *Plasmodium* infection. As demonstrated, with samples under 800 parasites/μL the system could trigger false-negative results (6%). Considering WHO guidelines in microscopy diagnosis, a parastitaemia of 80–200 parasites/μL are defined as difficult detection samples for certified WHO microscopists. Therefore, our system performance would be acceptable, although it should be evaluated following WHO microscopy diagnosis guidelines in clinical validation studies. RDTs might have a higher sensitivity and could also complement the final diagnosis (Slater et al., 2022).

Parasite density estimations by thick blood smear samples are performed following CDC recommendations to obtain autonomous calculations (CDC – DPDx – Diagnostic Procedures – Blood Specimens, n.d.). Parasite levels are crucial in *Plasmodium* infection and could determine the severity of malaria disease; therefore, leukocyte labeling is important and could provide valuable descriptive diagnostic information.

Descriptive metrics (precision, recall, *F*-score) can be compared with other predictive models based on CNNs for malaria parasite detection. When thick blood smear algorithms were compared with our predictive models, in most cases, *F*-score values (0.92–0.93) were very similar to the state-of-the-art reported in the literature (Yang et al., 2020; Kassim et al., 2021). In addition, a remarkable characteristic of our database is the heterogeneity of samples/images from different laboratories, preparation procedures, staining, smartphone/integrated camera acquisition, and *Plasmodium* species. Most studies used samples/images from a single laboratory or a single *Plasmodium* specie, commonly *P. falciparum*. In contrast, the addition of different images in terms of visual differences would affect the final descriptive parameters of the algorithm, although it would confer robustness to detect diverse preparations (Maron et al., 2021). Thin blood smear algorithms for parasite detection usually have higher values of precision, recall and, consequently, *F*-score, when compared with thick blood smears (Loddo et al., 2022; Magotra and Rohil, 2022). In addition, the customization of CNNs to improve detection results is generating optimal algorithms, such as the REONet method (modified ResNet-18) to classify malaria parasites on thin blood smears with 96.68% specificity, 94.79% sensitivity, and a 95.69% *F*-score (Zhu et al., 2022). This fact would explain the higher values of descriptive parameters in that type of study. Another important aspect was the comparative study of different object-detection CNNs. As demonstrated, the neural network model is crucial to obtain a reliable diagnosis, and the different structures and processing of each one would determine the final results. The same dataset was evaluated with different object detection CNNs, demonstrating optimal results with the YOLOv5x and Faster R-CNN models. The most efficient neural network is YOLOv5, however, the recall values of the Faster R-CNN model are slightly higher, and may perform better for the detection of samples with low parasite concentrations. In our study, YOLOv5x has the highest *F*-score value (92.79%) in comparison with other evaluated CNNs and attention modules, and is the most balanced neural network in terms of descriptive metrics. In addition, it is the model that best fits with our technology, as the processing and analysis of the images are fast (Li et al., 2022), and allows it to be integrated into the software of smartphone devices (Liu et al., 2022). By analyzing the metrics, speed and applicability of the YOLOv5x neural network, we could determine that it is the most suitable option for our system. However, the Faster R-CNN model could be a similar alternative with comparable results, at the expense of the RetinaNet and the SSD models. In the medical field, the minimization of false positive and negative results is an added value for the diagnosis. The neural network with the best sensitivity and recall, and consequently *F*-score, is the one that will generate the lowest errors. This factor reaffirms the choice of the YOLOv5x model for our system. Recent studies demonstrated that modification by increasing the feature scale and adding detection layers to the YOLOv3 and YOLOv4 algorithms could be an optimal solution to improve their performance in thick blood smear images (Abdurahman et al., 2021). Modified YOLOv4 obtains a mAP value of 96.32% for the detection of early trophozoites in thick blood smear digital images. When compared with our database results, their performance is slightly better, although mature trophozoites were not included for training and detection. Finally, single category results demonstrate that the detection of leukocytes is superior (*F*-score 0.990) when compared with early and mature trophozoites (*F*-scores of 0.886 and 0.873 respectively). YOLOv5x is demonstrated to be better with the detection of larger objects in digital images, as our results confirmed and in concordance with other studies (Liu et al., 2022). SE and CBAM attention modules were applied to YOLOv5x model, although they did not improve its final performance as demonstrated in statistical analysis. However, precision values of CBAM and recall values of SE were slightly better when compared to YOLOv5x, leading to a lowest ratio of false-positive and false-negative results, respectively. As an alternative to CNNs, there are transformer-based methods such as Detection Transformer (DETR) which are a transformer encoder-decoder architecture and a set-based global loss that forces unique predictions via bipartite matching for object detection (Carion et al., 2020).

It is important to note that the descriptive metrics of the neural networks evaluate the models generated and determine the final development validation of the whole prototype, however, they do not provide information on the sensitivity and specificity of the entire diagnostic system. Image auto-focusing issues, illumination changes, staining artifacts, or microscope model could affect the quality of the acquired images, therefore CNN prediction values would be negatively affected. In order to reliably evaluate the complete diagnostic tool, clinical validation tests should be pursued in reference laboratories and resource-poor settings. However, an earlier development phase, such as the one presented in this study, is crucial to provide the basis for future validation and implementation studies.

In addition, one of the key added values of the project is the universal microscope automation by means of the movement of the slide and the auto-focusing of the sample. The low-cost mechanism allows us to automate the process from beginning to end. A recent study demonstrated that the automation of a microscopic system for malaria detection could provide valuable results (Yoon et al., 2021). Yoon et al. (2021), developed a system with 100% sensitivity and specificity for the detection of *P. falciparum* cultures and *P. vivax* samples (Yoon et al., 2021). Lower-resolution images or microscope models could explain our different results, however, our novel diagnostic system is affordable, easy to use, universally adaptable, cheap, and specifically designed for any type of laboratory and infrastructure. Portable solar batteries provide an alternative to relying on electrical power to operate our system. Other design proposals for robotization and implementation based on a 3D-portable mobile microscope are of significant value to this area of study, and help improve and advance the development of portable and automated diagnostic systems (García-Villena et al., 2021).

Moreover, several studies demonstrated the utility of smartphone devices for the automatic diagnosis of malaria parasites via imaging techniques (Cesario et al., 2012; Poostchi et al., 2018; Yang et al., 2020; Yu et al., 2020; Zhao et al., 2020). Its powerful analog, digital, and telecommunication functions, combined with cloud data processing, confer smartphones with a wide range of diagnostic possibilities (Merazzo et al., 2021). Yu et al., 2023 evaluated the performance of a smartphone-based malaria diagnostic application in thick blood smear images with promising results, and could be considered as a milestone for further studies (Yu et al., 2023). However, parasite levels were not evaluated as distinct from our system.

The validation and implementation of the *iMAGING* prototype in resource-poor setting environments would be the next step. The continuous loss of microscopist experts is a major problem for clinical laboratories, although microscopy should remain a reference method of high relevance in microbiological diagnosis (Bradbury et al., 2022). Therefore, the system was designed to be a supportive tool for microscopists, in order to facilitate their routinely laboratory practice for malaria diagnosis and could be a suitable option for their training, by the continuous visualization of *Plasmodium* parasites in digital microscopy images. It is crucial to understand traditional microscopy to validate and implement novel diagnostic techniques based on AI. The direct visualization of parasites by microscopic observation of blood smears is an unequivocal sign of a positive diagnosis, one of the major strengths of this procedure compared with molecular or RDT techniques (Bradbury et al., 2022).

## 5 Conclusion

Automated malaria diagnosis is a major challenge to improve and support traditional microscopic techniques. Artificial intelligence diagnostic techniques would not only be useful for implementation in malaria-endemic countries but also for professional training, sample digitization, and diagnostic support for any laboratory, regardless of their resources. It is only a matter of time before novel diagnostic techniques based on AI and image digitalization erupt into medical environments to provide support for traditional microscopy. Microscopic visualization of thick blood smears can be tough and complex, therefore, assisted diagnostic methods based on AI, such as the one described herein, could be a suitable supportive tool of great potential. The automation of the entire process by the robotization of conventional optical microscopes provides added value to the diagnostic system. The possibility of completely emulating traditional microscopy with its *X*-*Y* slide movements and sample autofocus issues is a major challenge. The system has great potential, however, it needs to be refined and validated in different laboratories to evaluate its performance in clinical practice. Furthermore, comparison with other diagnostic techniques already established and regulated for malaria diagnosis, such as conventional optical microscopy, RDTs, and PCR, would allow AI-based diagnostic techniques to be more accurately positioned within the currently available set of malaria diagnostic techniques. The diagnostic system described in this study, has significant value due to the automation of the process, the design of the prototype, the automated calculation of parasite density, and the support which it provides to conventional microscopy. However, the detection system would be optimized in terms of object detection, and the algorithms for differentiating the *Plasmodium* species should be implemented to provide a complete diagnosis in further studies.

In conclusion, we are ever closer to developing an AI-based diagnostic method for malaria parasite detection. Recent advances and improvements in convolutional neural network models confer a promising future for this type of methodology. The development of an effective automated diagnostic system with AI technology for malaria diagnosis is still a great challenge. Therefore, the coalescence of the fully-automated system via auto-focus and slide movements and the autonomous detection of *Plasmodium* parasites in digital images with a smartphone software and AI algorithms confers the prototype the optimal features to join the global effort against malaria, neglected tropical diseases and other infectious diseases of poverty.

## Data availability statement

The original contributions presented in the study are included in the article/Supplementary files, further inquiries can be directed to the corresponding authors.

## Ethics statement

This study was conducted in accordance with the Declaration of Helsinki and approved by the Clinical Research Ethics Committee (CEIm) of the Vall d’Hebron University Hospital/Vall d’Hebron Research Institute with reference number PR(AG)40/2023.

## Author contributions

CM, EC, and JJ-M: conceptualized and drafted the manuscript, wrote the manuscript, and designed the figures. AO provided advice about artificial intelligence topics and designed the figures. FS and ES provided continuous intellectual feedback on malaria diagnosis and the protocols used nowadays. All authors have revised the manuscript and provided valuable feedback. EC and JJ-M edited and revised the overall manuscript. All authors agreed to be accountable for the content of the work. All authors contributed to the article and approved the submitted version.
