# Supplementary material for: iMAGING: a novel automated system for malaria diagnosis by using artificial intelligence tools and a universal low-cost robotized microscope
Source: Front Microbiol. 2023 Nov 24;14:1240936. doi: 10.3389/fmicb.2023.1240936 (PMC10704928; doi:10.3389/fmicb.2023.1240936)
Supplement: Supplementary file 1 [file Table_1.DOCX]

| **Object-detection CNN model compared with attention modules** | **Test dataset** | | | | |
| --- | --- | --- | --- | --- | --- |
|  | **Precision** | **Recall** | **F-score** | **mAP0.5** | **p - value** |
| **YOLOv5x** | 0.9210 | 0.9350 | **0.9279** | 0.9440 | - |
| **YOLOv5x - CBAM** | **0.9350** | 0.9170 | 0,9259 | 0.9420 | 0.779 |
| **YOLOv5x – SE** | 0.9040 | **0.9380** | 0.9207 | **0.9450** | 0.348 |

**Supplementary Table 1.** Comparative table of object-detection YOLOv5x model with SE and CBAM attention modules performances. Descriptive parameter values of Precision, Recall, F-score, and Mean Average Precision (mAP0.5) are represented for Test dataset. YOLOv5x: You Only Look Once version 5 model x, SE: Squeeze and Excitation, CBAM: Convolutional Block Attention Module. Statistical analysis (paired t-test) to compare the performance of CNN and attention modules was performed (*p-value*<0.05).

**Empirical tests with other databases**

To empirically check if the trained YOLOv5x neural network for malaria parasite detection can adequately perform in other acquisition setups, an open-source image database was employed and analyzed. The Institute of Electrical and Electronic Engineers (IEEE) malaria thick blood smear dataset by F. Yang *et al*., 2021 was used as an external image sample database (Malaria Thick Blood Smears | IEEE DataPort, n.d.). All images (3024×4032-pixel resolution) were acquired with a smartphone camera. To emulate a real clinical analysis with our system, images were cropped as in our pre-processing protocol. Finally, the YOLOv5x trained model was executed to analyze and detect leukocytes and parasites in IEEE digital images. Qualitative detection results are shown in **Supplementary** **Figure 2**. The neural network presented positive results with images from other databases.

**References**

Malaria Thick Blood Smears | IEEE DataPort (n.d.). Available at: https://ieee-dataport.org/documents/malaria-thick-blood-smears [Accessed November 15, 2021].
