# Supplementary figures and images for: iMAGING: a novel automated system for malaria diagnosis by using artificial intelligence tools and a universal low-cost robotized microscope
Source: Front Microbiol. 2023 Nov 24;14:1240936. doi: 10.3389/fmicb.2023.1240936 (PMC10704928; doi:10.3389/fmicb.2023.1240936)

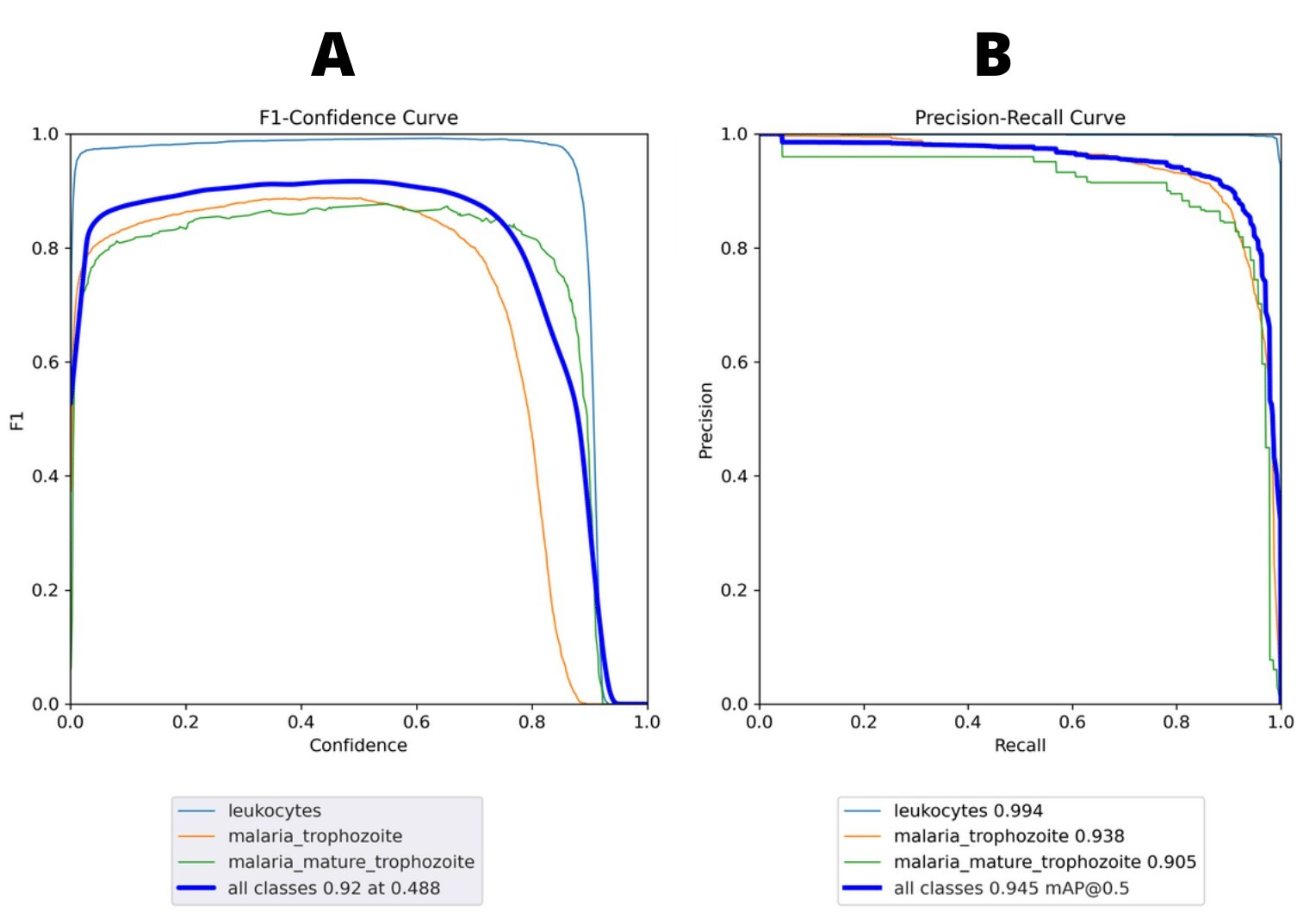

Supplement: Supplementary file 2 [file Image_1.TIFF]

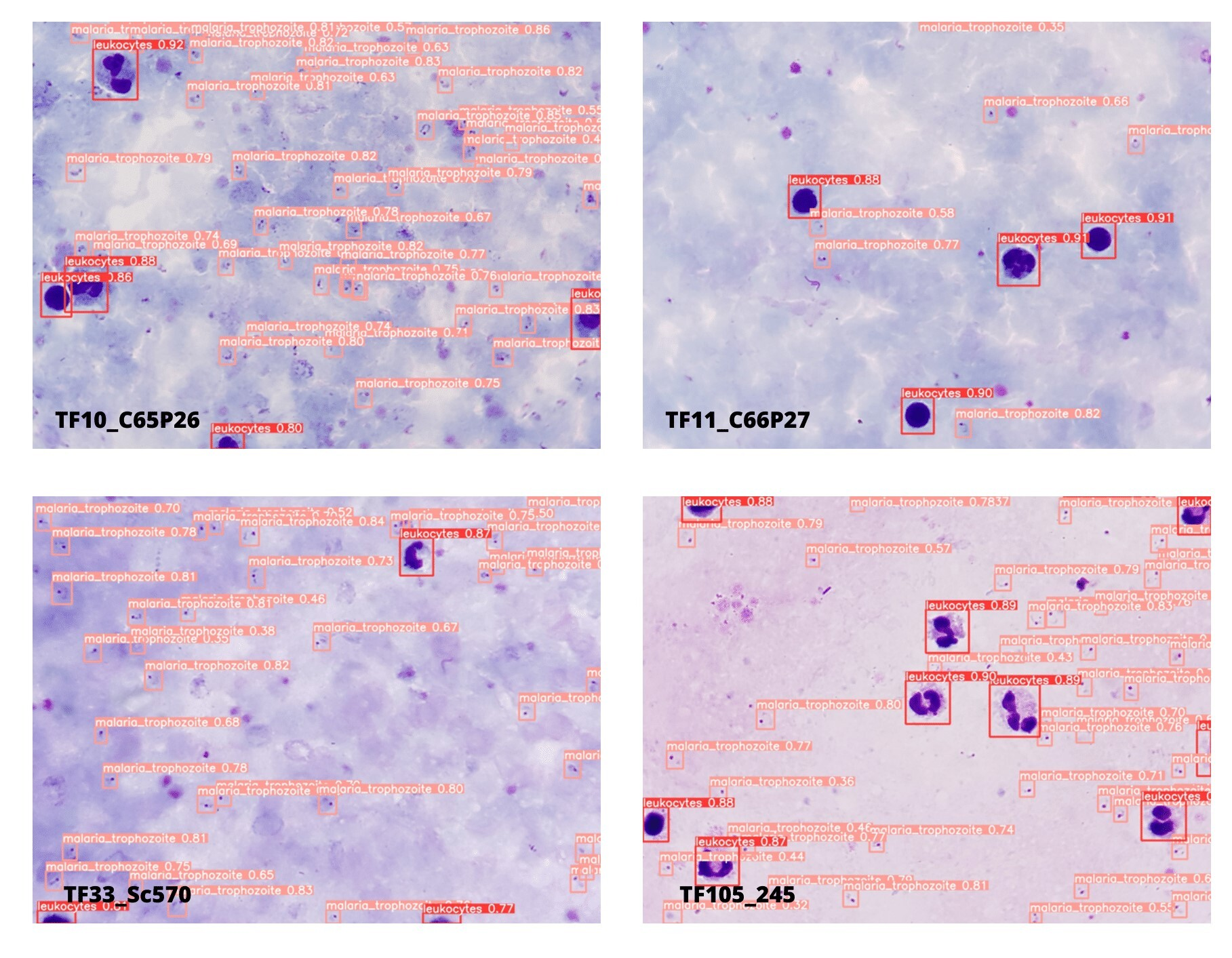

Supplement: Supplementary file 3 [file Image_2.TIFF]
